# Cost risk benefit analysis to support chemoprophylaxis policy for travellers to malaria endemic countries

**DOI:** 10.1186/1475-2875-10-130

**Published:** 2011-05-17

**Authors:** Eduardo Massad, Ben C Behrens, Francisco AB Coutinho, Ronald H Behrens

**Affiliations:** 1 School of Medicine, University of São Paulo and LIM 01 HCFMUSP, Brazil; 2 London School of Hygiene and Tropical Medicine, UK; 3 Dept of Economics University of Sheffield, UK

## Abstract

**Background:**

In a number of malaria endemic regions, tourists and travellers face a declining risk of travel associated malaria, in part due to successful malaria control. Many millions of visitors to these regions are recommended, via national and international policy, to use chemoprophylaxis which has a well recognized morbidity profile. To evaluate whether current malaria chemo-prophylactic policy for travellers is cost effective when adjusted for endemic transmission risk and duration of exposure. a framework, based on partial cost-benefit analysis was used

**Methods:**

Using a three component model combining a probability component, a cost component and a malaria risk component, the study estimated health costs avoided through use of chemoprophylaxis and costs of disease prevention (including adverse events and pre-travel advice for visits to five popular high and low malaria endemic regions) and malaria transmission risk using imported malaria cases and numbers of travellers to malarious countries. By calculating the minimal threshold malaria risk below which the economic costs of chemoprophylaxis are greater than the avoided health costs we were able to identify the point at which chemoprophylaxis would be economically rational.

**Results:**

The threshold incidence at which malaria chemoprophylaxis policy becomes cost effective for UK travellers is an accumulated risk of 1.13% assuming a given set of cost parameters. The period a travellers need to remain exposed to achieve this accumulated risk varied from 30 to more than 365 days, depending on the regions intensity of malaria transmission.

**Conclusions:**

The cost-benefit analysis identified that chemoprophylaxis use was not a cost-effective policy for travellers to Thailand or the Amazon region of Brazil, but was cost-effective for travel to West Africa and for those staying longer than 45 days in India and Indonesia.

## Background

Up to 37,000 European travellers acquired malaria during 1999 [1]. In 2005, 28 million U.S. travellers visited countries endemic for malaria and 856 cases were reported in U.S. civilians to the National Malaria Surveillance System. The risk of malaria in American travellers was calculated to vary between 137.3 per 10^5 ^travellers, during visits to Africa, to a risk of malaria of 0.012 per 10^5^, during visits to Mexico. The risk during a visit to South America was estimated at 2.12 per 10^5 ^visits [2]. Over the period 1989-99 in Europe, 680 people died from infection with *Plasmodium falciparum*, for which the use of chemoprophylaxis is highly effective in preventing disease [1].

In the absence of a vaccine, chemoprophylaxis is the only reliable method for preventing malaria in travellers. Five regimens are commonly available to prevent infection: mefloquine, doxycycline, atovaquone/proguanil, chloroquine, and under special circumstances, primaquine [3].

A significant number of travel-associated malaria cases are attributable to failure to adhere to the recommended chemoprophylaxis regimens. The proportion of UK residents who visit malaria endemic countries and use chemoprophylaxis has been reported at around 50% [4].

Although chemoprophylaxis with a drug such as mefloquine is highly effective at preventing malaria all drugs are known to cause adverse reactions, which demands their prescribing to be carefully targeted [5]. Chemoprophylaxis would be recommended to individuals at risk of falciparum malaria, where the risk of infection is higher than the risk of suffering a severe adverse event(s) [6]. Malaria chemoprophylaxis is selected based on an individual's risk assessment of the traveller, the safety and efficacy of the chemoprophylatic regimen(s), drug resistance, and the malaria transmission intensity in the region to be visited [7]. This is usually supported by a national recommendation using a similar analysis. Policy ideally should contain a cost-benefit component to supplement the above risk-assessment.

In this paper, a framework using a partial cost-benefit analysis was created to decide where a policy of recommending malaria prophylaxis would be economically rational. A partial cost-benefit analysis was employed as there are incommensurable and intangible costs for example "pain and suffering" and loss of leisure, that cannot be easily quantified monetarily and are excluded from the analysis.

This framework is applied to analyse the cost-effectiveness of malaria chemoprophylaxis policy. The analysis undertaken within this paper will not investigate the costs and benefits from one singular perspective, such as society or health systems view, which in the UK, bears the costs of the treatment of malaria, or the individual traveller, who bears both personal costs, purchasing prophylaxis drugs, lost earnings when suffering malaria and a loss of leisure cost when experiencing adverse events from chemoprophylaxis. Rather this paper will analyse the overall effectiveness of malaria chemoprophylaxis policies; irrespective of the distribution of the associated costs and benefits.

The framework is adjusted for probability of malaria transmission (malaria risk), duration of visit and cost of providing chemoprophylaxis in the UK, and includes the cost of adverse events and the societal costs of treatment of malaria in the UK. This model allowed the calculation of a threshold risk of malaria, below which the costs of prescribing and taking chemoprophylaxis is greater than the costs of avoided malaria. Areas with variable transmission intensity were selected to investigate the relationship of duration of exposure and risk of malaria infection. The Amazon region of Brazil was compared to popular tourist countries with similar higher and lower transmission intensity.

### The model

The model has three components: a probability component; a cost component; and a malaria risk component.

### Probabilities

**Figure 1:**
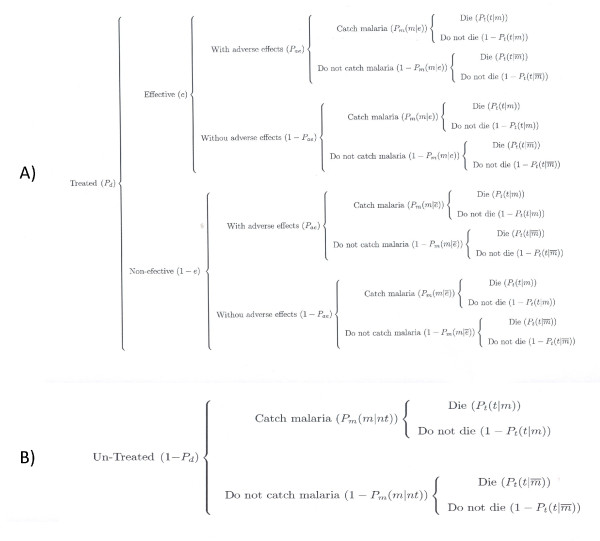
**Probability space for the Treated (a) individual and non-treated (b)individuals**.

An individual traveller to a malaria endemic area is considered to face two options either receiving or not chemoprophylaxis. Those called 'treated' receive chemoprophylaxis with a probability *P*_*d*_, whereas those called 'un-treated' do not receive chemoprophylaxis with probability (1 - *P*_*d*_) Treated individuals can be either protected, with probability (efficacy of the drug) *e*, or not protected (poor compliance and breakthrough), with probability (1 - *e*). Both protected and unprotected individuals can be either subject to adverse effects, with probability *P*_*ae*_, or not, with probability (1 - *P*_*ae*_). For the sake of generality we consider the possibility that both treated and un-treated can acquire malaria. We denote *P*_*m*_(*m *| *e*) and  the probabilities that a treated individual gets malaria (*P*_*m*_(*m *| *e*)), and the probability that a poorly compliant treated individual gets malaria (). The complementary probabilities of not acquiring malaria are (1 - *P*_*m*_(*m *| *e*)) and , respectively. Individuals who acquire malaria can either die of it, with probability *P*_*t*_, or not, with probability (1 - *P*_*t*_). The two outcomes for an individual who does not acquire malaria is they either die from other causes or survive, with conditional probabilities explained below. Un-treated individuals, in turn, can either acquire malaria or not, with likely probabilities. In addition, un-treated individuals can either die or not, with the correspondent probabilities. The theoretical space of probabilities considered is summarized in Figure 1.

Figures 1a and 1b show the conditional probabilities for treated individuals who acquire malaria (or not) depending on their protection status, that is, *P*_*m*_(*m *| *e*) for those whose treatment was considered effective, or , for those whose treatment was considered ineffective. In addition, we consider that the probabilities of dying as conditional to the fact of having malaria or not, that is *P*_*t*_(*t *| *m*) or . Similarly, un-treated individuals can acquire malaria or not, with conditional probabilities *P*_*m*_(*m *| *nt*) and (1 - *P*_*m*_(*m *| *nt*)), and dying or not with the similar conditional probabilities as treated individuals.

### Costs

The costs components included in the analysis are the cost of chemoprophylaxis, denoted *C*_*c*_; the cost of developing adverse effects, denoted *C*_*ae*_; the cost of acquiring malaria, denoted *C*_*m*_; the cost of dying of malaria, denoted *C*_*t*_; the theoretical cost of dying of other causes rather than malaria, denoted *C*_*o*_; and the cost of avoiding chemoprophylaxis, denoted *C*_*nc*_.

Therefore, the total cost of chemoprophylaxis has 16 components (Figure 1a), and the total cost of avoiding chemoprophylaxis has four components (Figure 1b), summarized in Table 1, and detailed in table 2. We used the price of the most expensive regimen, atovaquone/proguanil chemoprophylaxis in the model. Other less expensive regimens could be used to explore policy options.

**Table 1:** Costs components.

Cost of Prophylaxis	Cost of adverse event	Cost of Malaria
Travel^1^	Loss of leisure^1^	Loss leisure^1^
Assumed an approx. journey cost across 3 zones in London.	The cost of the lost days of holiday depending on severity of the reaction.	The cost of the lost days of holiday depending on severity of the reaction.
Prescription^1^	Medical costs^1^	Medical costs^3^
Cost of Atovaquone/Proguanil from a pharmacy.	A severe level of adverse event requires addition medical consultation.	Cost of NHS hospitalization for malaria severity adjusted.
health costs^3^		Economic & Social costs^2^
Nurses consult cost.		Includes days of work lost and social cost of death. The social cost of death encapsulates the average discounted value of remaining years of life coming solely from consumption and earnings.
Social costs^2^ Work time lost to society.		

1: Costs are born by the traveller

2: Costs born by society

3: Costs are born by the NHS

**Table 2:** Breakdown of the monetary values.

Costs considered for prescribing chemoprophylaxis include:	
Travel costs	£6
Prescription costs	£44
2 hours off work [14]	£24.48
Cost of Atovaquone/Proguanil from pharmacy	£41
Nurse consult cost [15]	£22.33
Adverse events and their costs are divided in three levels of severity:	
Mild event	
Holiday time loss (1 days) [16]	£164.29
Moderate event	
Holiday time loss (3 days) [16]	£492.86
Severe event	
Holiday time loss (7 days) [16]	£1,150
Hospital costs (3 days) [15]	£8,088
Illness Costs are divided into three levels of severity:	
Mild Malaria	
Personal costs (7 lost work days) [14]	£459
Days in Hospital (3 days) [15]	£8,088
Serious Malaria	
Personal costs (14 lost work days) [14]	£459
Days in Hospital (7 days) [15]	£18,872
Death from Malaria	
Social cost of death [17-19]	£312,000
The social cost of death is calculated as the average discounted value of remaining years of life coming from UK consumption and earnings. Assumed to have a 5% discount rate	
Age 55 Life expectancy is 78. Average income of £27,000 therefore social cost is £312,000	

Therefore, the total cost of chemoprophylaxis is the sum of the costs  and the total cost of avoiding chemoprophylaxis is the sum of the costs, with *C*_*i *_as in additional file 1.

### The risk of acquiring malaria

The probability of acquiring malaria in Brazil *P*_*m *_was calculated per year from the number of imported cases from Brazil to the UK divided by the number of visits made to Brazil by UK residents. We compared this probability of infection (risk) to UK travellers to India, Thailand, Indonesia and West Africa (Ghana, Nigeria and Sierra Leone) identified as non-index countries The denominator for rates was the number of UK residents visiting the countries. This is collated from overseas travel by UK residents obtained from the Office for National Statistics (ONS), collected as part of the International Passenger Survey (IPS). The IPS is a year round survey of incoming and outgoing passengers' at all major ports. Around 250,000 face to face interviews of a randomly selected sample of passengers (representing 0.2% of all travellers) provides estimates of the total annual visits made by UK residents to other countries. The number of imported cases was obtained from malaria surveillance reports collected by the Malaria Reference Laboratory (MRL) part of the Health Protection Agency (HPA). The MRL, as the national reference laboratory, obtains enhanced passive surveillance reports of malaria cases through laboratories and clinicians. The risk of malaria was estimated as the incidence per visit. The numerator was the number of cases reported each year to the MRL.

Transmission of malaria in the five regions was assumed to be homogeneous.. However, if seasonality is important, this variation could be incorporated into the model in a similar manner as used in the Brazilian Amazon [8].

### Cost-benefits analysis

Cost-benefits analysis is based on the assumption that whenever *S*_1 _<*S*_2 _a policy supporting the use of chemoprophylaxis is worthwhile; and the other way around, whenever *S*_1 _>*S*_2 _chemoprophylaxis in not cost effective overall. In addition, there is a threshold in the risk of acquiring malaria  such that, for a given cost profile (that is, the values assumed for the costs *C*_1_, i = 1,...,20), that risk is greater than the threshold, chemoprophylaxis is worthwhile. This threshold is found by making *S*_1 _= *S*_2_.

Before proceeding with the calculation of the threshold in malaria transmission intensity, it is important to note that some of the probabilities involved in the costs *C*_1 _(i = 1,...,20) are equal, or very close to zero, namely *P*_*m *_(*m *| *e*) and  (as a consequence, the cost of mortality by other causes, *C*_*o *_is also considered zero). This reduces the number of costs to be computed from 20 to 11. The remaining costs are *C*_4_, *C*_8_, *C*_9_, *C*_10_, *C*_12_, *C*_13_, *C*_14 _and *C*_16 _for the treated individuals and *C*_17_, *C*_18 _and *C*_20 _for the non-treated individuals. Therefore, making *S*_1 _= *S*_2 _with the remaining costs we can estimate  as:(1)

where Π, Δ, Ω, and Ξ as in additional file 2.

### Illustrating the model

In order to illustrate the theory above, a malaria endemic area of the Amazonian region in Brazil as used. The total number of endemic malaria cases has annually oscillated around 600,000 over the past decade [9], of which one quarter are *P. falciparum *infections [8].

This region was used to test the model and estimate the expected risk of a traveller acquiring malaria dependent on the exposure duration. We used an established model described by Massad *et al *[8].

**Figure 2:**
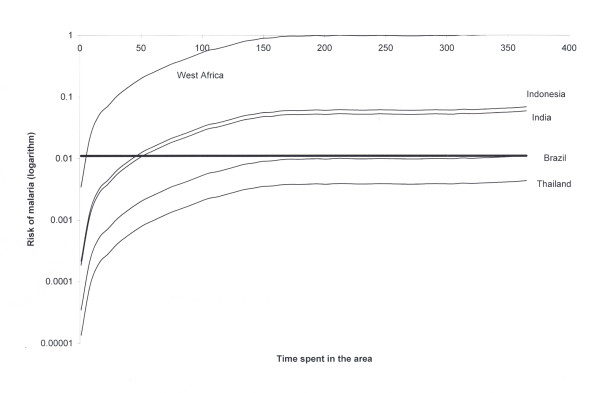
**Risk of acquiring malaria for travellers to the Brazilian Amazon region as a function of the time spent in the area.** Travellers are assumed to arrive at summer time, the season with the highest transmission (see 8)..

The highest probability of a traveller acquiring malaria in the Brazilian Amazon region is during the summer and is demonstrated as a function of the duration of exposure in Figure 2.

The risk of acquiring all malaria (falciparum and non-falciparum) for travellers to all the study regions and countries, assuming that the incidence density (force of infection) is approximately equal to the probability of acquiring malaria (see additional file 3). Utilizing the model [8] the relative risk of malaria for UK travellers to each of those regions was compared to that calculated for the Amazon region of Brazil, set as equal to one. Additional file 4 details the imported cases and the number of UK arrivals to the regional countries.

Assuming the costs and probabilities components of equation (1) and shown in table 3, a cost-benefit threshold  for malaria was  = 0.011275 (1.13%).

**Table 3:** Costs and probabilities components for the calculation of.

*Cost/Prob.*	*Value*	*Source*
*C*_*c *_the cost of chemoprophylaxis	£138.00^#^	[14]
*C*_*ae *_cost of adverse events	£164.29^+^	[14,15,17]
*C*_*m *_cost of acquiring malaria	£18,872.00*	[15,16]
*C*_*t *_cost of dying of malaria	£312,000	[17]
*C*_*nc *_cost of avoiding chemoprophylaxis	0	--
*P*_*d *_probability of using chemoprophylaxis	0.5	[20]
*P*_*ae *_probability of adverse effect	0.4	[21]
*P*_*t *_probabilityof dying from malaria	0.0004	[22]
*e *efficacy of prophylaxis	0.9	[23]

Costs are expressed in Sterling Pounds

^#^sum of the costs as in table 2, ^+^cost of mild reaction, *cost of serious malaria (see appendix 4 for details).

**Figure 3:**
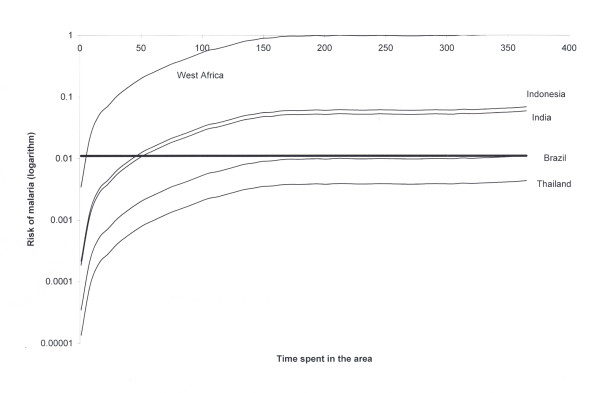
**Logarithm of the risk of acquiring malaria for travellers to 5 regions, compared with the Brazilian Amazon region**. The cost-benefit threshold is the thick horizontal line (see equation 1). Confidence intervals were omitted for the sake of clarity.

With this as the accumulated risk, the number of days exposure a traveller needs to remain in a region to achieve this threshold risk was calculated and shown in Figure 3 and Table 4.

**Table 4:** Relative incidence of malaria for Brazil as compared with other countries.

Region/Country	Relative incidence (95% C.I.)	Time in days to threshold 1.13% (95% C.I.)
Index: Brazil (Amazon)	1.0	>365
West Africa	99 (86-112)	30 (26-34)
India	5.3 (4.9-5.7)	53 (49-57)
Indonesia	6.17 (4.4-7.9)	45 (32-57)
Thailand	0.39 (0.35-0.43)	>365

Duration of exposure in days each to reach the cost-benefit-risk threshold

Travellers to Brazil and Thailand never achieve the minimal threshold whatever their duration of visit. The higher the probability of malaria, the shorter the exposure period to reach the cost-benefit threshold (thick horizontal line).

Travel data for the countries analysed can be seen in additional file 4.

In terms of policy on choice of chemoprophylaxis during visits to Brazil and Thailand, it would not be cost-effective to recommend chemoprophylaxis whatever the duration of visit. The threshold is most rapidly reached in West Africa (~1 month) and is greater than 45 days in visits to India and Indonesia.

## Discussion

In this paper, the estimated cost-benefit threshold for policy on the use of malaria chemoprophylaxis for British travellers visiting a variety of malaria endemic regions was calculated. The analysis was based on available cost data, covering direct and indirect costs of using malaria prophylaxis, incorporating for the first time, the cost of adverse events associated with the use of chemoprophylaxis and the health and treatment cost associated with acquiring malaria.

If the direct and indirect costs of using chemoprophylaxis would fall from our models £138.00 to £15.00, the cost-benefit threshold would fall from 1.12% to 0.044%. The influence of the cost of the chemoprophylaxis when reduced can have an important impact on the risk threshold. Using alternative agents such as mefloquine or doxycycline can change this cost component. However, this does note change the main results of our analysis (see additional file 5 for the calculations with alternative agents). The study threshold level of 1.13% is higher than that described by Behrens and Roberts [4], whose cost-benefit analysis of malaria chemoprophylaxis for travellers concluded that overall costs of preventing malaria in travellers exceed costs of providing chemoprophylaxis. They argued chemoprophylaxis was cost effective and worthwhile when the malaria incidence was 0.7% per visit for an approximate visit of 14 days in travellers from the UK. The authors applied similar direct and indirect cost variables as in our model.

The travellers malaria probability is based on malaria imported into the UK against the numerator of visits by UK residents to five regions/countries. This malaria probability is likely to be subject to reporting bias, and has not been adjusted for chemoprophylaxis usage or numbers of truly exposed travellers (regional travel), so may not represent a true attack rate. We have also used all species of malaria, and if *Plasmodium vivax *infections had been excluded, on the ground that chemoprophylaxis is only useful for falciparum malaria, the difference between Africa and remainder of the regions would have been significantly greater. For visitors to West Africa, Behrens *et al *[10,11] estimated the malaria incidence in returning travellers as 0.14%-0.26%. per visit. For travellers to S-E Asia the incidence in Thailand, Cambodia and Vietnam was calculated to be <1 case per 100,000 visits (0.001% per visit) an incidence well below (1.13%) the cost effectiveness threshold for chemoprophylaxis policy. The model considers the risk of acquiring malaria as a function of the duration of exposure (visit). Using the transmission intensity estimated from the probability of infection, an individual traveller's benefit from chemoprophylaxis could be adjusted using the duration of exposure, taking into account the calculated threshold

Usually cost-benefit analysis takes a perspective of one group in the analysis. This analysis was based around rationalising a healthy policy perspective, which included the societal burden of the treatment of malaria, the individual traveller, bearing the costs of purchasing prophylaxis, lost earnings when ill and loss of leisure when experiencing adverse events from chemoprophylaxis. The policy could also consider an endemic country perspective where reducing the requirement for chemoprophylaxis for visitors to the region/country makes it more attractive to visitors and increases financial gain. It is also feasible that such a policy based on transparent and evidence based data may encourage travellers to accept policy recommendation and adhere to and complete their prescribed chemoprophylaxis regimen when it is rationally based. This is particularly relevant when travellers can observe a policy which is influenced by duration of exposure. Policy would be more believable where 1 week and 1-year visit require different prophylaxis regimens.

These findings must be interpreted within the limitations of the model and data quality. The model considers only economic costs, and does not include pain and suffering, but includes values for time lost through both adverse events and illness from malaria. This analysis should be considered as a methodological proposal rather than a recipe for decision-making. The model is very dependent on the actual incidence of travellers' malaria to the regions and on the precision of the cost estimates for each component of the model

The model is flexible enough to adjust for intensity of transmission. For a different set of costs and probabilities than those in Table 2, the model will allow the estimated cost-benefit calculation and hence the optimal chemoprophylaxis policy for travellers to any malarious area. The incidence of malaria in the visited area is critical, as the cost-benefit ratio is very sensitive to changes in the intensity of malaria transmission. Where there are changes in transmission through seasonality, malaria control interventions, or epidemic outbreaks this will have a significant bearing on the model.

Growing international travel [12] and the anxiety and concerns over the side effects of chemoprophylaxis, which can affect up to 30% [13] of users, complicates policy recommendations for low malaria risk regions. The study reveals that at higher malaria probabilities (in part due to longer exposure), the cost-benefit calculation supports the recommendation of chemoprophylaxis. However, where the risk of malaria acquisition varies from region to region, a more detailed cost analysis can lead to rational recommendations and selection of appropriate preventative measures.

Based on the estimated incidence in UK travellers, an average visit time of 25 days, and a set of cost parameters, this study suggests chemoprophylaxis policy would not be cost-effective for UK visitors travelling to Brazil or Thailand but would be cost-effective for visits to West Africa and longer visits to India and Indonesia

## Competing interests

The authors declare that they have no competing interests.

## Authors' contributions

EM and RB designed the study. EM and FABC created the probability model. RB collected, analysed and interpreted UK malaria and travel data and contributed to the final manuscript. BCB collected and analysed the economic and cost model and all contributed to the final manuscript.

All authors read and approved the final manuscript.

## Supplementary Material

Additional file 1 **Costs components of the model**. Total costs of chemoprophylaxis Click here for file

Additional file 2 **Costs components of the model**. Components of equation (1) of the main text Click here for file

Additional file 3 **Deduction of the probability of malaria**. How the probability of getting malaria is identified with the incidence Click here for file

Additional file 4 **Travel data**. Travel data for the countries analysed. Click here for file

Additional file 5 **Model's results with different chemoprophylaxis agents**. Duration of exposure in days each to reach the cost-benefit-risk threshold for three different chemoprophylaxis agents Click here for file
