# Supplementary material for: Cost risk benefit analysis to support chemoprophylaxis policy for travellers to malaria endemic countries
Source: Malar J. 2011 May 17;10:130. doi: 10.1186/1475-2875-10-130 (PMC3123601; doi:10.1186/1475-2875-10-130)
Supplement: Additional file 1 — Costs components of the model. Total costs of chemoprophylaxis [file 1475-2875-10-130-S1.DOC]

Additional file 1

File format: DOC

Title: Costs components of the model

Description: Total costs of chemoprophylaxis

| **Total costs of chemoprophylaxis.** | |
| --- | --- |
| **Treated Individuals** | |
| $C_{1}$ | $(C_{c}+C_{ae}+C_{m}+C_{t})P_{d}eP_{ae}P_{m}(m\vert e)P_{t}(t\vert m)$ |
| $C_{2}$ | $(C_{c}+C_{ae}+C_{m})P_{d}eP_{ae}P_{m}(m\vert e)(1-P_{t}(t\vert m)$ |
| $C_{3}$ | $(C_{c}+C_{ae}+C_{o})P_{d}eP_{ae}(1-P_{m}(m\vert e))P_{t}(t\vert\overline{m})$ |
| $C_{4}$ | $(C_{c}+C_{ae})P_{d}eP_{ae}(1-P_{m}(m\vert e))(1-P_{t}(t\vert\overline{m}))$ |
| $C_{5}$ | $(C_{c}+C_{m}+C_{t})P_{d}e(1-P_{ae})P_{m}(m\vert e)P_{t}(t\vert m)$ |
| $C_{6}$ | $(C_{c}+C_{m})P_{d}e(1-P_{ae})P_{m}(m\vert e)(1-P_{t}(t\vert m))$ |
| $C_{7}$ | $(C_{c}+C_{o})P_{d}e(1-P_{ae})(1-P_{m}(m\vert e))P_{t}(t\vert\overline{m})$ |
| $C_{8}$ | $C_{c}P_{d}e(1-P_{ae})(1-P_{m}(m\vert e))(1-P_{t}(t\vert\overline{m}))$ |
| $C_{9}$ | $(C_{c}+C_{ae}+C_{m}+C_{t})P_{d}(1-e)P_{ae}P_{m}(m\vert\overline{e})P_{t}(t\vert m)$ |
| $C_{10}$ | $(C_{c}+C_{ae}+C_{m})P_{d}(1-e)P_{ae}P_{m}(m\vert\overline{e})(1-P_{t}(t\vert m))$ |
| $C_{11}$ | $(C_{c}+C_{ae}+C_{o})P_{d}(1-P_{m}(m\vert\overline{e})P_{t}(t\vert\overline{m})$ |
| $C_{12}$ | $(C_{c}+C_{ae})P_{d}(1-e)P_{ae}(1-P_{m}(m\vert\overline{e})(1-P_{t}(t\vert\overline{m}))$ |
| $C_{13}$ | $(C_{c}+C_{m}+C_{t})P_{d}(1-e)(1-P_{ae})P_{m}(m\vert\overline{e})P_{t}(t\vert m)$ |
| $C_{14}$ | $(C_{c}+C_{m})P_{d}(1-e)(1-P_{ae})P_{m}(m\vert\overline{e})(1-P_{t}(t\vert m)$ |
| $C_{15}$ | $(C_{c}+C_{o})P_{d}(1-e)(1-P_{ae})(1-P_{m}(m\vert\overline{e}))P_{t}(t\vert\overline{m})$ |
| $C_{16}$ | $C_{c}P_{d}(1-e)(1-P_{ae})(1-P_{m}(m\vert\overline{e}))(1-P_{t}(t\vert\overline{m}))$ |
| **Un-treated Individuals** | |
| $C_{17}$ | $(C_{nc}+C_{m}+C_{t})(1-P_{d})P_{m}(m\vert nt)P_{t}(t\vert m)$ |
| $C_{18}$ | $(C_{nc}+C_{m})(1-P_{d})P_{m}(m\vert nt)(1-P_{t}(t\vert m))$ |
| $C_{19}$ | $(C_{nc}+C_{o})(1-P_{d})(1-P_{m}(m\vert nt))P_{t}(t\vert\overline{m})$ |
| $C_{20}$ | $C_{nc}(1-P_{d})(1-P_{m}(m\vert nt))(1-P_{t}(t\vert\overline{m}))$ |
