# Supplementary material for: Cost risk benefit analysis to support chemoprophylaxis policy for travellers to malaria endemic countries
Source: Malar J. 2011 May 17;10:130. doi: 10.1186/1475-2875-10-130 (PMC3123601; doi:10.1186/1475-2875-10-130)
Supplement: Additional file 2 — Costs components of the model. Components of equation (1) of the main text [file 1475-2875-10-130-S2.DOC]

Additional file 2

File format: DOC

Title: Costs components of the model

Description: Components of equation (1) of the main text

Costs components of equation (1) of the main text:

$$\Delta=(C_{c}+C_{ae})P_{d}eP_{ae}+C_{c}P_{d}e(1-P_{ae})+(C_{c}+C_{ae})P_{d}(1-e)P_{ae}+C_{c}P_{d}(1-e)(1-P_{ae})$$

$$\begin{matrix} \Omega=(C_{c}+C_{ae}+C_{m}+C_{t})P_{d}(1-e)P_{ae}P_{t}(t|m)+(C_{c}+C_{ae}+C_{m})P_{d}(1-e)P_{ae}(1-P_{t}(t|m) \\ +(C_{c}+C_{ae})P_{d}(1-e)P_{ae}+(C_{c}+C_{m}+C_{t})P_{d}(1-e)(1-P_{ae})P_{t}(t|m) \\ +(C_{c}+C_{m})P_{d}(1-e)(1-P_{ae})(1-P_{t}(t|m))+C_{c}P_{d}(1-e)(1-P_{ae}) \end{matrix}$$

$$\Pi=C_{nc}(1-P_{d})$$

$$\Xi=(C_{nc}+C_{m}+C_{t})(1-P_{d})P_{t}(t|m)+(C_{nc}+C_{m})(1-P_{d})(1-P_{t}(t|m))$$
