# Supplementary material for: Cost risk benefit analysis to support chemoprophylaxis policy for travellers to malaria endemic countries
Source: Malar J. 2011 May 17;10:130. doi: 10.1186/1475-2875-10-130 (PMC3123601; doi:10.1186/1475-2875-10-130)
Supplement: Additional file 3 — Deduction of the probability of malaria. How the probability of getting malaria is identified with the incidence [file 1475-2875-10-130-S3.DOC]

Additional file 3

File format: DOC

Title: Deduction of the probability of malaria

Description: How the probability of getting malaria is identified with the incidence

In this appendix we demonstrate how the probability of getting malaria can be approximately identified with the per capita incidence (force of infection) of the disease.

Let *a* be the number of bites a mosquito inflicts in a human population per unit time. Let also *m* be the total number of infected mosquitoes divided by the total human population and *b* the probability that an infectious bites produces a new human case. Then in a small time interval *dt* the number of susceptible individuals (*N_S_*) acquiring malaria is $abmN_{S}dt$. Therefore the per capita incidence (force of infection) in a given region is give by

$i=abm$ (A1)

Assume now that a small number of travelers *V_0_* arrive at the region at time W. The number of those travelers that are still uninfected at time W+w, *V*(W+w) is given by

$V(\Omega+\omega)=V_{0}\exp(-\int_{\Omega}^{\Omega+\omega} abmdt)$ (A2)

So, the probability of getting infected in this region during the period between W and W+w is

$p(\Omega,\omega)=1-\exp(-\int_{\Omega}^{\Omega+\omega} abmdt)\cong\int_{\Omega}^{\Omega+\omega} abmdt$ (A3),

which is the total number of new cases per capita in the period between W and W+w . Since we are interested in the annual incidence, regardless of the transmission be homogeneous throughout the year we may choose W as an arbitrary day and w as equal to 1 year. We then have that the annual per capita incidence of malaria ($\int_{\Omega}^{\Omega+1} abmdt$) in that region is approximately equal to the probability of acquiring malaria in that year.
