# Supplementary material for: Cost risk benefit analysis to support chemoprophylaxis policy for travellers to malaria endemic countries
Source: Malar J. 2011 May 17;10:130. doi: 10.1186/1475-2875-10-130 (PMC3123601; doi:10.1186/1475-2875-10-130)
Supplement: Additional file 4 — Travel data. Travel data for the countries analysed. [file 1475-2875-10-130-S4.DOC]

Additional file 4

File format: DOC

Title: Travel data

Description: Travel data for the countries analysed.

Travel data for the countries analysed.

|  | **India** | | **Thailand** | | **West Africa** | | **Brazil** |  | **Indonesia** | |
| --- | --- | --- | --- | --- | --- | --- | --- | --- | --- | --- |
| average duration of visit (d) | 25 |  | 24 |  | 28 |  | 23 |  | 25 | |
|  | visits | cases | visits | cases | visits | cases | visits | cases | visits | cases |
| 2003 | 496,919 | 51 | 278,937 | 2 | 126,235 | 443 | 57,432 | 2 | 13,766 | 6 |
| 2004 | 656,718 | 68 | 362,883 | 4 | 172435 | 427 | 71,156 | 2 | 34,766 | 2 |
| 2005 | 795,592 | 91 | 355,858 | 0 | 200,166 | 455 | 85,800 | 2 | 28,126 | 1 |
| 2006 | 913,721 | 91 | 411,208 | 1 | 195,657 | 486 | 112,930 | 4 | 37,540 | 5 |
| 2007 | 971,537 | 105 | 404,156 | 0 | 237,470 | 433 | 98,456 | 4 | 41,725 | 2 |
| 2008 | 956,470 | 119 | 394,743 | 2 | 252,615 | 429 | 132,644 | 2 | 53,614 | 0 |
| 2009 | 847,000 | 99 | 338,000 | 2 | 270,000 | 455 | 219,000 | 1 | 20,101 | 3 |

Visits by UK residence from IPS survey

Average duration of visit from IPS survey

Reported cases of all malaria to MRL
