# Supplementary material for: Cost risk benefit analysis to support chemoprophylaxis policy for travellers to malaria endemic countries
Source: Malar J. 2011 May 17;10:130. doi: 10.1186/1475-2875-10-130 (PMC3123601; doi:10.1186/1475-2875-10-130)
Supplement: Additional file 5 — Model's results with different chemoprophylaxis agents. Duration of exposure in days each to reach the cost-benefit-risk threshold for three different chemoprophylaxis agents [file 1475-2875-10-130-S5.DOC]

Additional file 5

File format: DOC

Title: Model’s results with different chemoprophylaxis agents

Description: Duration of exposure in days each to reach the cost-benefit-risk threshold for three different chemoprophylaxis agents

| **Duration of exposure in days each to reach the cost-benefit-risk threshold for three different chemoprophylaxis agents** | | | |
| --- | --- | --- | --- |
|  | **Time in days to threshold** | | |
| **Region/Country** | **Atovaquone/proguanil**  **(£41.00)** | **Mefloquine**  **(£14.00)** | **Doxycycline**  **(£2.00)** |
| Index: Brazil | > 365 | 157 | 149 |
| West Africa | 30 | 30 | 25 |
| India | 53 | 45 | 43 |
| Indonesia | 45 | 43 | 40 |
| Thailand | > 365 | > 365 | > 365 |
